# Genetics of blood malignancies among Iranian population: an overview

**DOI:** 10.1186/s13000-020-00968-2

**Published:** 2020-05-06

**Authors:** Majid Ghayour-Mobarhan, Amir Sadra Zangouei, Seyed Mohammad Hosseinirad, Majid Mojarrad, Meysam Moghbeli

**Affiliations:** 1 Metabolic Syndrome Research Center, School of Medicine, Mashhad University of Medical Sciences, Mashhad, Iran; 2 Student Research Committee, Faculty of Medicine, Mashhad University of Medical Sciences, Mashhad, Iran; 3 Department of Medical Genetics and Molecular Medicine, School of Medicine, Mashhad University of Medical Sciences, Mashhad, Iran

**Keywords:** Blood, Malignancy, Risk factor, Panel marker, Iran

## Abstract

**Background:**

Blood malignancies are among the leading causes of cancer related deaths in the world. Different environmental and genetic risk factors are involved in progression of blood malignancies. It has been shown that the lifestyle changes have affected the epidemiological patterns of these malignancies. Hematologic cancers are the 5th common cancer among Iranian population. It has been observed that there is a rising trend of blood malignancies incidences during the recent decades. Therefore, it is required to design novel diagnostic methods for the early detection of such malignancies in this population.

**Main body:**

In present review we have summarized all of the significant genes which have been reported among Iranian patients with blood malignancies. The reported genes were categorized based on their cell and molecular functions to clarify the molecular biology and genetics of blood malignancies among Iranian patients.

**Conclusion:**

It was observed that the epigenetic and immune response factors were the most frequent molecular processes associated with progression of blood malignancies among Iranian population. This review paves the way of introducing a population based panel of genetic markers for the early detection of blood malignancies in this population.

## Background

**Fig. 1:**
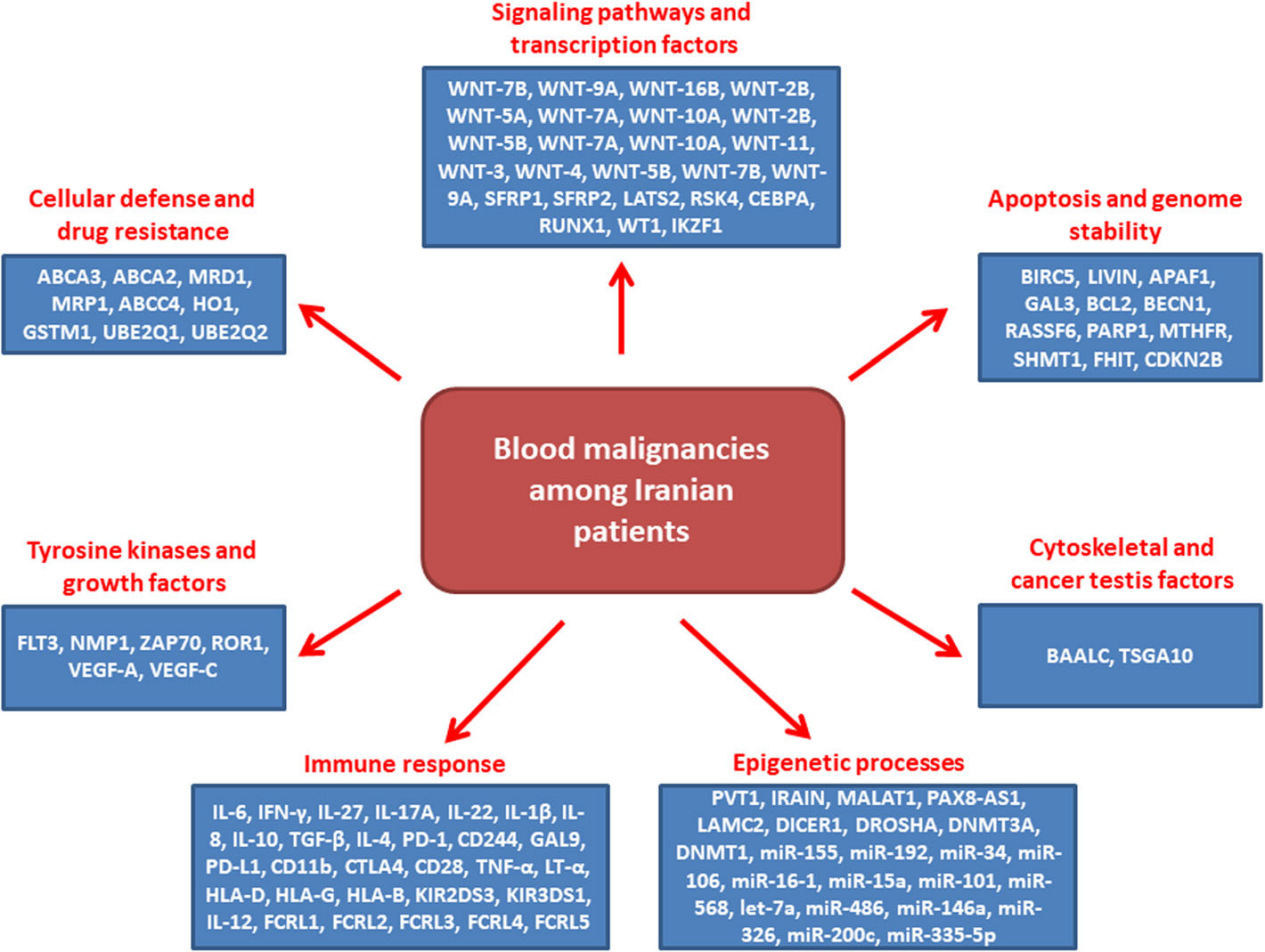
All of the genes which are involved in progression of blood malignancies among Iranian population

Cancer is an important health challenge in all countries regardless of economic status which is the leading cause of mortality in developed countries and the second cause of death in developing countries [1]. Blood malignancies involve about 10% of all newly diagnosed cases in the U. S and almost 8% of newly diagnosed cancers in Europe [2, 3]. It has been reported that there was 176,200 new cases of blood cancers in 2019 in the U. S [3]. Blood malignancies can be categorized into the leukemia, lymphoma, and plasma cell disorders which originate from bone marrow and lymphatic system [2]. Leukemia is associated with incomplete maturation and excessive proliferation of white blood cells in the blood or bone marrow [4]. Based on cell types and disease course, the leukemia is categorized into four major subgroups including acute lymphoblastic leukemia (ALL), acute myeloid leukemia (AML), chronic myeloid leukemia (CML), and chronic lymphoblastic leukemia (CLL). AML is the most frequent type (25%) of adult leukemia [5] with the lowest survival rate among all leukemias [6]. ALL is also the most frequent (about one-third) childhood blood malignancy [7]. Moreover, it involves about 80% of the leukemias among children [4]. CML accounts for 15% of adult leukemia which is more predominant among males [8, 9]. CLL is the most common type of leukemia in western countries and is mainly observed in elderly [10]. Lymphoma is characterized by clonal proliferation of lymphocytes and is categorised into Hodgkin Lymphoma (HL) and non-Hodgkin Lymphoma (NHL) with 10 and 90% of the cases, respectively [11, 12]. Multiple myeloma (MM) is a plasma cell malignancy involving about 10% of all hematological malignancies [13, 14]. In recent years, industrialization and modernity have changed lifestyles and environments that affected the epidemiological patterns of different types of disease and cancers [15]. Although, the etiology of blood malignancies is still under debate, various risk factors such as chemicals exposure, radiation, tobacco, economical status, viruses, and genetic defects have been suggested for blood malignancies [16–21]. There is a noticeable ratio of blood malignancies among Iranian population in which the leukemia involves about 8% of total cancer cases in Iran. The most common types are ALL, AML, and CLL which are higher in men compared with women [22]. Different risk factors such as blood groups, familial history, drug usage during pregnancy, occupation, radiation, smoking, magnetic fields, and Epstein-Barr virus infection are associated with leukemia progression in Iran [23–26]. Since, the majority of patients refer for treatment in advanced stages of blood malignancies, it is required to introduce novel molecular early detection methods to decrease the high ratio of mortality in such patients. Regarding the high incidence of blood malignancies among Iranian population, in present review we summarized all of the significant reported genes in different blood malignancies which have been reported until now among Iranian patients to pave the way of introducing a population based panel of genetic markers (Table.1). Moreover, we categorized all of the reported genes based on their cell and molecular functions to clarify the molecular biology of blood malignancies in this population for the first time in the world (Fig. 1).

**Table 1:** All of the factors involved in blood malignancies among Iranian population

STUDY (ET AL)	Year	Type	Gene	population	Results	Prediction
izadifar [27]	2018	AML	PVT1	134 patients 40 controls	Over expression in AML-M3	Diagnosis
pashaiefar [28]	2018	AML	IRAIN	64 patients 51 controls	Under expression.	Poor prognosis
ahmadi [29]	2018	CLL	MALAT1	30 patients 30 controls	Over expression.	Diagnosis
bahari [30]	2018	ALL	PAX8-AS1	110 patients 120 controls	Polymorphism was correlated with tumor progression.	Diagnosis
hashemi [31]	2016	ALL	Lnc-LAMC2	110 patients 120 controls	Polymorphism was correlated with tumor progression.	Diagnosis
koolivand [32]	2018	AML	miR-155	25 patients 25 controls	Over expression.	Poor prognosis
fathullahzadeh [33]	2016	CLL	miR-192	20 patients 20 controls	Under expression.	Diagnosis
hashemi [34]	2016	ALL	miR-34b/c	110 patients 120 controls	Polymorphism was correlated with tumor progression.	Diagnosis
fallah [35]	2015	CML	miR-155, miR-106, miR-16-1, miR-15a, miR-101, and miR-568	50 patients	MiR-155 and miR-106 under expressions. MiR-16-1, miR-15a, miR-101, and miR-568 over expressions.	Diagnosis and prognosis
seyyedi [36]	2016	AML	miR-1, miR-486, and let-7a	45 patients	Over expressions.	Diagnosis
hasani [37]	2014	ALL	miR-146a	75 patients 115 controls	Polymorphism was correlated with tumor progression.	Diagnosis
hashemi [38]	2017	ALL	DROSHA	75 patients 115 controls	Polymorphism was correlated with tumor progression.	Diagnosis
farzaneh [39]	2016	CLL and ALL	DICER	51 patients 29 controls	Under expression.	Diagnosis
rahmani [40]	2017	ALL	DNMT1	45 patients	Methylation.	Poor prognosis
allahbakhshian [41]	2018	ALL	IL-6 and IFN-γ	52 patients 13 controls	IFN-γ under expression in ALL. IL-6 under expression in T-ALL.	Diagnosis
ghavami [42]	2018	ALL	IL-27	200 patients 210 controls	Polymorphism was correlated with tumor progression.	Poor prognosis
kouzegaran [43]	2018	CLL	IL-17A and IL-22	78 patients 28 controls	Over expressions.	Diagnosis
sepehrizadeh [44]	2014	AML	IL-1β, IL-8, IL-10, and IFN-γ	46 patients	IL-1β, IL-8, and IL-10 under expressions after chemotherapy. IFN-γ over expression after chemotherapy.	Diagnosis
amirzargar [45]	2005	CML	TGF-β, IL-4, and IL-10	30 patients 40 controls	TGF-β over expression. IL-4 and IL-10 under expressions.	Diagnosis
abdolmaleki [46]	2018	AML	PD-1 and CD244	30 patients 15 controls	Over expressions.	Diagnosis
taghiloo [47]	2017	CLL	GAL-9 and PD-L1	25 patients 15 controls	Over expressions.	Poor prognosis
amirghofran [48]	2001	AML	CD11b	70 patients	Correlation with survival.	Diagnosis
ramzi [49]	2018	AML, ALL, CML	CTLA-4, CD28, and PD-1	59 patients 46 controls	Polymorphisms were correlated with tumor progression.	Diagnosis
nasiri [50]	2013	ALL, NHL	TNF-α and LT-α	138 patients 130 controls	Polymorphisms were correlated with tumor progression.	Diagnosis
orouji [51]	2012	ALL	HLA-D	135 patients 150 controls	Allele frequencies were correlated with tumor progression.	Diagnosis
rezvany [52]	2007	CLL	HLA-G	74 patients 12 controls	Over expression.	Diagnosis
amirzargar [53]	2007	CML	HLA-D	50 patients 80 controls	Allele frequencies were correlated with tumor progression.	Diagnosis
khosravi [54]	2007	CML	HLA-D	50 patients 180 controls	Allele frequencies were correlated with tumor progression.	Diagnosis
sarafnejad [55]	2006	AML	HLA-D	60 patients 180 controls	Allele frequencies were correlated with tumor progression.	Diagnosis
moazzeni [56]	1999	CLL	HLA-B and HLA-D	32 patients	Allele frequencies were correlated with tumor progression.	Diagnosis
shahsavar [57]	2010	AML and ALL	KIR2DS3	78 patients 200 controls	Genotype frequencies were correlated with tumor progression.	Diagnosis
noori-daloii [58]	2013	GVHD	IL-1α, IL-4Rα, and IL-12	91 patients	Polymorphisms were correlated with tumor progression.	Good prognosis
kazemi [59]	2009	ALL	FCRL1–5	73 patients 35 controls	Under expressions.	Diagnosis
pouyanrad [60]	2019	ALL	miR-335-5p	64 patients 30 controls	Under expression.	Poor prognosis
rahgozar [61]	2014	ALL	ABCA3, ABCA2, MRD1, and MRP1	27 patients 15 controls	Were correlated with drug resistance.	Poor prognosis
mahjoubi [62]	2008	AML and ALL	MRP1	52 patients 10 controls	Correlation with poor clinical outcomes.	Poor prognosis
mahjoubi [63]	2012	ALL	MRP1	42 patients 10 controls	Over expression.	Poor prognosis
ghodousi [64]	2018	ALL	miR-326 and miR-200c	46 patients 16 controls	Under expressions.	Poor prognosis
mesrian tanha [65]	2017	ALL	ABCC4	145 patients	Polymorphism was correlated with tumor progression.	Poor prognosis
kazemi [66]	2018	Leukemia	HO-1	63 patients	Genotype frequencies were correlated with tumor progression.	Good prognosis
saadat [67]	2000	ALL	GSTM1	38 patients 75 controls	Allele frequencies were correlated with tumor progression.	Diagnosis
seghatoleslam [68]	2014	ALL	UBE2Q1	20 patients 20 controls	Under expression.	Diagnosis
seghatoleslam [69]	2012	ALL	UBE2Q2	20 patients 20 controls	Over expression.	Diagnosis
zareifar [70]	2018	AML	Livin and BIRC5	43 patients	Correlation with poor prognosis.	Poor prognosis
rostami [71]	2017	AML	APAF1	101 patients 50 controls	Methylation.	Diagnosis
asgarian-omran [72]	2010	CLL	GAL-3	85 patients	Under expression.	Poor prognosis
daneshbod [73]	2005	AML	BCL-2	70 patients	Correlation with survival.	Poor prognosis
zare-abdollahi [74]	2016	AML	BECN1	128 patients	Under expression.	Prognosis
amirghofran [75]	2009	ALL	BCL-2	50 patients	Correlation with poor prognosis.	Poor prognosis
younesian [76]	2017	ALL	RASSF6	45 patients	Methylation.	Poor prognosis
pashaiefar [77]	2018	AML	PARP1	65 patients 54 controls	Over expression.	Poor prognosis
pashaiefar [78]	2018	AML	PARP1	78 patients 19 controls	Over expression.	Poor prognosis
bahari [79]	2016	ALL	MTHFR	100 patients 120 controls	Polymorphism was correlated with tumor progression.	Diagnosis
bahari [80]	2016	ALL	SHMT1	120 patients 120 controls	Polymorphism was correlated with tumor progression.	Diagnosis
bahari [81]	2017	ALL	FHIT	100 patients 120 controls	Methylation.	Diagnosis
kamali dolatabadi [82]	2017	AML	CDKN2B	59 patients	Methylation.	Diagnosis
memarian [83]	2012	ALL	WNT-7B, WNT-9A, WNT-16B, WNT-2B, WNT-5A, WNT-7A, WNT-10A	71 patients 36 controls	WNT-7B, WNT-9A, and WNT-16B over expressions. WNT-2B, WNT-5A, WNT-7A, and WNT-10A under expressions.	Diagnosis
memarian [84]	2009	CLL	WNT-3, WNT-4, WNT-5B, WNT-7B, WNT-9A, WNT-10A, WNT-16B, WNT-7A	62 patients 11 controls	WNT-3, WNT-4, WNT-5B, WNT-7B, WNT-9A, WNT-10A, and WNT-16B over expressions. WNT-7A under expression.	Diagnosis
memarian [85]	2007	AML	WNT-3, WNT-7A, Wnt-10A	16 patients 36 controls	WNT-3 over expression. WNT-7A and Wnt-10A under expressions.	Diagnosis
ghasemi [86]	2015	AML	SFRP1, SFRP2	43 patients 25 controls	Methylation.	Diagnosis
gholami [87]	2014	AML	LATS2	32 patients 10 controls	Over expression.	Poor prognosis
rafiee [88]	2016	AML	RSK4	40 patients 10 controls	Under expression.	Poor prognosis
salarpour [89]	2017	AML	CEBPA, RUNX-1	96 patients 18 controls	CEBPA and RUNX-1 over expressions.	Diagnosis
ayatollahi [90]	2017	AML	WT1	126 patients	Over expression.	Diagnosis
rezai [91]	2015	Leukemia	WT1	12 patients 12 controls	Over expression.	Diagnosis
asgarian omran [92]	2008	ALL	WT1	116 patients 36 controls	Over expression.	Diagnosis
bahari [93]	2016	ALL	IKZF1	110 patients 120 controls	Polymorphism was correlated with tumor progression.	Diagnosis
rezaei [94]	2017	AML	FLT3, NPM1	70 patients	Mutation.	Diagnosis
nasiri [95]	2014	AML	FLT3	27 patients	Mutation.	Diagnosis
pazhakh [96]	2011	AML	NPM1	131 patients	Mutation.	Diagnosis
zaker [97]	2010	AML	FLT3, KIT	212 patients	Mutation.	Diagnosis
jafari ghahfarokhi [98]	2014	CLL	ZAP70	66 patients	Over expression.	Good prognosis
shabani [99]	2008	ALL	ROR1, WT1	51 patients	Over expressions.	Diagnosis
aliparasti [100]	2013	AML	VEGF-C	27 patients 28 controls	Under expression.	Diagnosis
amirpour [101]	2016	AML	BAALC	47 patients 47 controls	Over expression.	Poor prognosis
nadimi [102]	2016	ALL, AML	BAALC	145 patients	Polymorphism was correlated with tumor progression.	Poor prognosis
mobasheri [103]	2006	ALL	TSGA10	52 patients	Expression in ALL cases.	Diagnosis and prognosis
hoseinkhani [104]	2019	AML	TSGA10, HIF-1α	30 patients 10 controls	TSGA10 under expression. HIF-1α over expression.	Diagnosis

## Main text

### Non-coding RNAs, RNA processing, and methylation

Beside the genetic changes, the epigenetic alterations are also associated with tumor progression. DNA methylation, chromatin remodeling, and non-coding RNAs are the main epigenetic processes involving in neoplastic transformations [105]. Long non-coding RNAs (lnc-RNAs) are a class of non-coding RNAs (nc-RNAs) with transcripts of more than 200 nucleotides [106]. PVT1 is a lnc-RNA that induces angiogenesis via inhibition of miR-26b to activate the CTGF and ANGPT2 [107]. It has been reported that the AML-M3 cases had higher levels of PVT1 expression in comparison with healthy cases in a sample of Iranian subjects. High-risk AML-M3 cases had also higher levels of PVT1 expression compared with low- and intermediate-risk subjects. There were also higher levels of CCAT1 and CCDC26 expressions among AML-M4 and M5 patients compared with controls. Moreover, intermediate-risk patients had higher levels of CCDC26 compared with controls [27]. IRAIN is also a lnc-RNA that inhibits apoptosis and induces tumor cell proliferation by targeting LSD1 and EZH2 [108]. It has been reported that the IRAIN was significantly down regulated in a group of Iranian non-M3 AML patients compared with control group. They reported that the IRAIN was a poor prognostic factor in AML in which the patients with lower levels of IRAIN had shorter OS and RFS. They also introduced the IRAIN as a diagnostic marker with 70.3% specificity and 63% sensitivity. Moreover, the chemotherapeutic resistant cases had a low levels of IRAIN expression [28]. *MALAT1* is a lnc-RNA associated with alternative splicing and gene expression regulation. It promotes tumor cell proliferation via PI3K-AKT signaling pathway [109]. The expression level of MALAT1 was assessed in Iranian CLL patients compared with healthy controls. It was shown that the expression of MALAT1 was significantly higher in CLL group [29]. The PAX8 is an important transcription factor during embryonic development and maintaining normal tissue function [110]. PAX8 antisense RNA 1 (PAX8-AS1) is a lnc-RNA responsibe for the regulation of PAX8 [111]. It has been reported that the rs4848320 and rs6726151 variants of PAX8-AS1 significantly increased the risk of childhood ALL among a sub population of Iranian cases [30]. Another group assessed the impact of lnc-LAMC2–1:1 rs2147578 on risk of childhood ALL in a sample of Iranian cases. They showed that the CG, GG and CG + GG genotypes of lnc-LAMC2–1:1 significantly increased the risk of childhood ALL. Moreover, the rs2147578 G allele significantly increased the risk of childhood ALL compared with the C allele [31].

MicroRNAs (miRNAs) are a class of small (~ 20–22 nt) nc-RNAs that are involved in gene regulation by binding to the 3′UTR sequence of target genes. MiR-155 induces tumor cell migration and proliferation by targeting SOCS1 and MMP16 [112]. It has been observed that the miR-155 was involved in differentiation of myeloid and erythroid cells, and also was significantly up regulated among a group of Iranian AML patients [32]. The results were compatible with other studies which have shown that the miR-155 is associated with lymphoma and different types of cancer [113, 114]. MiR-192-5p inhibits tumor growth by targeting YY1 [115]. It has been shown that there was a significant decreased expression of miR-192 among a sub population of Iranian CLL cases compared with healthy individuals [33]. The miR-34 family consists of miR-34a-c. While miR-34a is encoded by its own transcript, miR-34b, and miR-34c have a primary transcript (pri-miR-34b/c). MiR-34 suppressess cell proliferation and invasion by targeting CD44 and NOTCH1 [116, 117]. The rs4938723 C > T polymorphism in promoter sequence of pri-miR-34b/c affects GATA-X binding and pri-miR-34b/c expression. It has been shown that the pri-miR-34b/c rs4938723 CC and TC + CC genotype significantly reduced risk of childhood ALL among Iranian cases [34]. MiR-106 promotes the tumor cell metastasis by targeting ALEX1 [118]. MiR-16-1 also suppresses the tumor cell proliferation through TWIST1 targeting [119]. The miR-15a and miR-101 reduce tumor cell growth through regulation of WNT and PI3K/AKT signaling pathways, respectively [120, 121]. It has been reported that there were miR-155 and miR-106 under expressions, while miR-15a, miR-101, miR-16-1, and miR-568 over expressions among a subpopulation of Iranian CML cases [35]. The miR-1, miR-486, and let-7a inhibit tumor cell growth by targeting EVI-1, FOXO1, and AURKB, respectively [122–124]. It has been shown that there was miR-1 up regulation in Iranian CN-AML patients compared with normal controls. Moreover, there was an association between miR-1 up regulation and NPM1 positive cases. The miR-486-5p and let-7a were also up regulated in CN-AML patients compared with healthy controls. Furthermore, they found increased let-7a expression in NPM1 positive CN-AML patients [36]. MiR-146a inhibits tumor cell proliferation and induces drug sensitivity by targeting SOD2 [125]. It has been shown that there was a significant correlation between hsa-miR-146a (rs2910164 G > C) polymorphism and increased childhood ALL susceptibility in a sample of Iranian subjects [37]. The ribonuclease III superfamily participates in RNA maturation and decay pathways [126]. Drosha is a class 2 ribonuclease III that performs the initiation step of miRNA processing in the nucleus [127]. It has been observed that the DROSHA rs642321 C > T variant significantly reduced the risk of ALL progression in dominant and codominant inheritance models. Moreover, the rs642321 T allele was protective compared with C allele in ALL cases among Iranians [38]. Dicer is also a key regulator in the miRNA biogenesis which is encoded by DICER1. It has been shown that the expression levels of the Dicer in patients with ALL and CLL were significantly lower than that in normal childs and adults, suggesting that the abnormal Dicer expression can be involved in ALL and CLL progressions among Iranian cases [39].

Beside the nc-RNAs, DNA methylation is also a critical process during the epigenetic regulation. DNMT3A catalyzes the methyl transfer to the CpG regions of DNA sequence [128]. The prognostic impact of DNMT3A and FLT3-ITD mutations were investigated among Iranian AML patients after HSCT. It was found that the FLT3-ITD was a poor prognostic indicator and overall survival of patients with DNMT3A mutations accompanied by FLT3-ITD that significantly reduced after hematopoietic stem cell transplantation (HSCT) [129]. Another study assessed methylation status of DNMT1 gene promoter sequence among a sample of Iranian ALL patients. It was observed that there was not any DNMT1 promoter methylation in T- and B-ALL patients, which may suggest a higher expression level of this gene in these patients. In contrast, DNMT1 was partially methylated in healthy controls and Pre B-ALL patients [40].

### Inflammatory factors and immune response

The immune system has a critical role against the tumor progression [130]. Interferon gamma (IFN-γ) and IL-6 are two important cytokines associated with immune responses against tumor cells. IL-6 has a dual function as pro-inflammatory and anti-inflammatory cytokine [131]. The expression levels of IL-6 and IFN-γ were assessed in a sub population of Iranian ALL patients which showed a significant reduction of IFN-γ in ALL patients compared with controls. Although, there was not any change in IL-6 expression level among B-ALL patients, it was reduced significantly among T-ALL patients compared with healthy controls [41]. The IL-12 family includes IL-12, IL-23, IL-27, and IL-35 which are involved in interactions between adaptive and innate immunity. It has been reported that there were significantly higher frequencies of IL-27 rs153109 AG genotype and G allele among a sample of Iranian ALL cases. There was also higher frequency of rs17855750 TG genotype and G allele in ALL patients. Moreover, genotype combination analysis illustrated a high prevalence of the rs153109 AG + GG and rs17855750 TG + GG genotypes, indicating that the rs153109 G and rs17855750 G carriers had high risk of ALL progression. Patients with rs153109 AG genotype and rs17855750 TG genotype had worse prognosis. The high frequency of G allele was correlated with poorer drug response among Iranian ALL patients [42]. IL-17 is a pro-inflammatory cytokine secreted by T helper 17 cells following IL-23 induction. It induces secretion of various cytokines such as IL-6, TNF-α, and IL-8. It has been reported that the PBMCs of CLL patients had higher levels of IL-17A and IL-22 mRNA expressions compared with healthy subjects. Moreover, the IL-17A and IL-22 plasma levels were significantly higher in CLL patients compared with control group among Iranians [43]. IL-1β is a pro-inflammatory cytokine that promotes the B cells and lymphocytes proliferation. The IL-1β is also involved in proliferation of myeloid progenitor cells through up regulation of colony-stimulating factor (CSF). IFN-γ is also inhibitor of the cell proliferation. IL-8 is an anti-inflammatory factor that plays important role as a chemoattractant for neutrophils during inflammation. IL-10 is an inhibitor of proinflammatory cytokines. G-CSF induces the proliferation and differentiation of neutrophils which is used to increase the neutrophil counts following chemotherapeutic treatments. It has been shown that the levels of IL-1β, IL-8, and IL-10 expressions were reduced and increased after chemotherapy and G-CSF treatment respectively, which introduced these cytokines as efficient molecular markers for AML chemotherapeutic monitoring in a sample of Iranian cases. IFN-γ was also up regulated after chemotherapy and down regulated after G-CSF treatment [44]. Genetic profile of Th1 and Th2 cytokines were assessed among a sub population of Iranian CML cases. It was observed that the most frequent genotypes were IFN-γ AT, TGF-β TG/TG, IL-4 CC − 590, and IL-10 ACC/ACC. Moreover, there were higher levels of TGF-β and lower levels of IL-4 and IL-10 in CML cases compared with normal subjects [45].

T cell exhaustion is an immunosuppressive mechanism of anti-tumor immune responses. Programed death 1 (PD-1), TIM-3, and CD244 are inhibitory receptors involved in regulation of T cell exhaustion. TIM-3 is an immune checkpoint and regulates CD8+ T-cell exhaustion through PD-1 [132]. It has been observed that there were significant increased expressions of PD-1 and CD244 on CD4+ and CD8+ T cells in Iranian AML patients compared with control group [46]. PD-L1 and GAL-9 are ligands of PD-1 and TIM-3, respectively. GAL-9 and PD-L1 were significantly over expressed in CLL patients compared with healthy cases. Moreover, their expressions were correlated with the expressions of their TIM-3 and PD-1 ligands. Furthermore, patients with higher clinical stages showed higher levels of GAL-9 and PD-L1 mRNA expressions. Therefore, GAL-9 and PD-L1 can be considered as two potential biomarkers of CLL prognosis and progression among Iranian cases [47]. CD11b is associated with leukocyte adhesion and migration during inflammatory responses. It has been reported that there were significant correlations between CD11b, complete remission duration, and survival among a sub population of Iranian AML cases [48].

CTLA-4 functions as an immune checkpoint to inhibit immune responses and is expressed in regulatory T-cells [133]. The inducible T-cell costimulator (ICOS) is also an immune checkpoint belonged to the CD-28 family which is expressed by activated T-cells and has a critical role in cell proliferation and immune response [134]. The association between CTLA-4, ICOS, and CD28 polymorphisms and leukemia risk were assessed among Iranian patients. It was observed that the frequencies of TT genotype of CTLA-4 -318 T/C, AA genotype of CTLA-4 + 49 A/G, and CT genotype of PD-1 1.9 C/T polymorphisms were significantly lower in patients compared with controls. Patients with leukemia showed significant increased frequencies in AG genotype of CTLA-4 + 49 A/G, CC genotype of PD-1 1.9 C/T, and the CT genotype of CD28 + 17C/T polymorphisms. The A allele of CTLA-4 + 49 A/G and CC genotype of CD28 + 17 C/T polymorphisms were markedly higher in cases with acute leukemia compared with chronic leukemia. Moreover, AML cases had a higher frequency of AA genotype of CTLA-4 + 49A/G, however, the AG genotype was more frequent among ALL cases [49]. NHL is a heterogeneous lymphoma which is categorized into diffuse large B-cell lymphoma (DLBCL) and follicular lymphoma (FL) subtypes. TNF-α and lymphtoxin-α (LT-α) are immunoregulatory cytokines associated with inflammation and apoptosis. It has been shown that there were significant differences of TNF-α-308 and LT-α + 252 polymorphisms among Iranian ALL and NHL patients, respectively [50].

The Human Leukocyte Antigen (HLA) is belonged to the major histocompatibility complex (MHC) and is associated with immune responses. The HLA-DR, DQ and DP genes are encoded by MHC II. It has been reported that the frequency of DQ5 allele carriers were similary increased among both of Iranian adult and childhood ALL cases. Moreover, there were increased and decreased DQ7 and DQ2 allele carriers in childhood ALL, respectively [51]. It has been reported that the DRB1*11 and DRB1*09 were the most and least frequent alleles in DRB1 locus among normal population. There was significant difference of DRB1*13 frequencies between patients and controls. It was concluded that the DRB1*13 was decreased and DRB1*04 was increased in Iranian ALL patients which can be suggested as protective and susceptible alleles during ALL progression, respectively [135]. HLA-G is a non-classical MHC-I antigen that is expressed in throphoblast, thymic epithelial cells, and cornea. It is associated with suppression of natural killer (NK) or T-cell apoptosis and inhibition of trans-endothelial migration of NK cells. It has been reported that the HLA-G was expressed in a sample of B-CLL patients, while there was not any expression in the healthy control group. It was concluded that the HLA-G affects immune response in B-CLL subjects [52]. Another study has been shown that there were significantly higher frequencies of DQB1*03011 and DQA1*0505, whereas significant lower frequency of DQB1*03032 among a sample of Iranian CML patients compared with controls. The frequencies of HLA-DRB1*07 and DQA1*0201 alleles were also higher in patients who were below 35 years old. Moreover, HLA-DRB1*11/−DQA1*03011/−DQB1*03011/−DQB1*0302/−DQA1*0505/−DRB1*04 were reported as the most frequent haplotypes among Iranian CML cases [53]. Another study has been shown that the presence of HLA-DRB1*11 allele increased the risk of AML, while HLA-DRB4 and –DQB1*0303 alleles were protective. Moreover, the HLA-DRB1*16 and HLA-DRB4 alleles predispose individuals to AML and CML, respectively in a sample of Iranian subjects [54]. Another study has been observed that there was a significant association between HLA-DRB1*11 and AML. The HLA-DRB4 and DQB1*0303 alleles were significantly less frequent in patients compared with controls. HLA-DRB1*11 allele increased the risk of AML while HLA-DRB4 and –DQB1*0303 alleles were protective against AML among a sample of Iranian subjects [55]. Another group reported that the frequencies of HLA-B13 and DR53 were significantly increased in Iranian B-CLL patients compared with healthy controls. However, patients showed decreased A11, B35, DR1, and Cw3 levels compared with control group which suggested their protective roles in CLL [56].

Natural killer cells (NK) are a subgroup of lymphocytes accounting for 10% of total peripheral blood lymphocytes. Killer cell immunoglobulin-like receptors (KIR) and their ligands (HLA-I), have an important function in regulation of NK cells. Decreased activity of NK cells as a result of inhibitory KIR-HLA state, causes the escape of leukemic cells from immunity. It has been reported that the AML cases had lower frequencies of KIR2DS3 and KIR3DS1 genotypes compared with controls among a sample of Iranian cases. There was also a high inhibitory KIR-HLA state in AML cases which was not observed in ALL patients [57]. Bone marrow transplantation (BMT) is an important therapeutic method for the malignant disorders [136]. However, it is restricted by acute graft-versus-host disease (aGVHD) which is the most common side effect of BMT. It was observed that the IL-4Rα + 1902, IL-1α-889, and IL-12–1188 polymorphisms were correlated with aGVHD among a sample of Iranian allogenic BMT recipients [58]. Fc receptor-like (FCRL) molecules are a family of Fc receptor homolog molecules mainly expressed by B lymphocytes. It has been observed that there were significant down regulations of FCRL1–5 in leukemic cells of a sub population of Iranian ALL patients [59].

### Cellular defense and drug resistance

Drug resistance is a big challenge of tumor therapy which is responsible for treatment failure in 90% of metastatic tumors. Various processes are involve in drug resistance such as drug transporters, DNA repair, and apoptosis [137]. ABC transporters are membrane proteins associated with drug efflux and resistance which can be regulated by non-coding RNAs. The miR-335-5p promotes tamoxifen resistance by targeting ERα signaling pathway [138]. It has been reported that the miR-335-3p expression level was lower in Iranian cALL patients compared with healthy controls. Moreover, ABCA3 up regulation was associated with lower levels of miR-335-3p expressions in cALL patients. The miR-335-3p was down regulated in drug resistant (MDR+) in comparison with drug sensitive (MDR-) cases. Moreover, NEAT1 and MALAT1 over expressions in cALL cases were associated with lower levels of miR-335-3p [60]. Another study has been observed that the ABCA3, ABCA2, MRD1, and MRP1 were significantly associated with drug resistance and increased risk of tumor relapse among a sub population of Iranian childhood ALL patients [61]. The MRP1 as a member of ABC-transporters is associated with anthracyclines, vincristine, and epipodophyllotoxins drug resistances. It has been shown that the MRP1 expression levels were significantly lower in Iranian remission AML cases compared with relapsed cases. The M5 subtype had significantly higher levels of MRP1 expression compared with other types. Moreover, There was a correlation between MRP1 expression and poor clinical outcomes [62]. Another study has also been found that the majority of Iranian pediatric ALL cases with relapse had MRP1 over expression, however there was not any correlation between the levels of MRP1 and clinicopathological features [63]. MiR-326 and miR-200c are associated with drug resistance through regulation of ABCC1 and PI3K/AKT signaling pathway, respectively [139, 140]. It has been shown that the miR-326 and miR-200c were down regulated in a sample of Iranian ALL patients compared with healthy subjects. There was an inverse association between the miR-326 and ABCA2 espressions. The miR-326 expression was reduced in MDR+ and relapsed ALL patients compared with MDR- group. Moreover, down regulation of miR-326 and miR-200c can be considered as biomarkers of childhood ALL among Iranians [64]. ATP-binding cassette subfamily C member 4 (ABCC4) is a highly polymorphic gene that exports organic anions and drugs form the cells. It is expressed in the apical or basolateral surface of hematopoietic cells and regulates the cAMP-dependent signal transduction [141]. A correlation between rs2274407 (G912T) polymorphism of ABCC4 and poor prognosis has been reported among a sample of Iranian pediatric ALL cases [65].

Heme Oxygenase-1 (*HO-1)* is a critical enzyme during heme metabolism and is associated with tumor resistance toward chemotherapy or radiotherapy-induced apoptosis. GT repeats (12–24 bp) in *HO-1* gene promoter adversely affect the basal promoter activity. Allelic frequencies of (GT)_n_ microsatellite polymorphisms in *HO-1* gene were compared between Iranian acute leukemia patients and healthy controls. The (GT)_n_ was categorized into short (S) alleles < 27 repeats and long (L) alleles ≥27 repeats. Three genotypes (SS, SL, and LL) were created for the study population. Although, allelic frequencies were not different between studied groups, there was significant higher frequency of “LL” genotype in leukemia patients with 3-years surveillance [66]. The human glutathione S-transferases (GSTs) are a family of detoxification enzymes which conjugate electrophilic substrates with glutathione. It has been observed that the null genotype of GSTM1 was significantly more frequent among Iranian ALL cases. Differences for cancer susceptibility in subjects may be related to the GSTM1 and CYP1A1 polymorphisms which are involved in the metabolism of carcinogens. The absence of GSTM1 alleles inhibits the detoxification of environmental carcinogens which can be resulted in tumorigenesis [67]. Ubiquitin proteasome system has also a critical function in cellular defence through degradation of aberrant and misfolded proteins. In this system, protein degradation starts with ubiquitin attachment through a series of enzymatic reactions including activating (E1), conjugating (E2), and ligating (E3). It has been reported that there was UBE2Q1 down regulation in majority of the leukemic samples which suggested its potential involvement during pathogenesis of ALL among Iranian patients [68]. Another study has been reported that there were higher levels of UBE2Q2 mRNA expressions in leukemic cells of ALL cases compared with healthy blood cells in a sample of Iranian cases [69].

### Apoptosis and genome stability

Apoptosis and DNA repair are the most important cellular processess involved in regulation of normal cell cycle and genome stability. Inhibitor of apoptosis proteins (IAPs) help cells to escape from apoptosis. Survivin (BIRC5) and livin are two IAPs which are associated with drug resistance. Association of survivin and livin with prognosis and survival were evaluated in Iranian pediatric AML cases which showed survivin and livin expression in majority of AML patients. There was significant direct association between survivin expression, risk of relapse, and high level of primary WBC. Livin and survivin were associated with poor prognosis of AML, although the results failed to determine them as independent prognostic factor [70]. Apoptotic protease activating factor 1 (APAF-1) is a tumor suppressor gene (TSG) which is involved in DNA damage-induced apoptosis. It is a key factor in intrinsic or mitochondrial pathway of apoptosis, which forms apoptosome in response to cytochrome c release [142]. The role of CpG islands promoter methylation of APAF1 was assessed in AML progression. It was reported that the cases with FLT3-ITD mutation had higher frequency of APAF1 promoter methylation compared with FLT3-wildtype patients. Moreover, the hyper methylated AML cases had decreased levels of APAF1 expressions compared with controls [71]. Galectins are a large family of lectins characterized by a carbohydrate recognition domain, which recognizes the β-galactoside structures. GAL-3 is mainly expressed in myeloid cells such as neutrophils, macrophages, and dendritic cells. It is involved in apoptosis regulation through BCL-2 [143]. It has been shown that there was a significant down regulation of GAL-3 in Iranian CLL patients compared with controls which suggested that as a suitable prognostic marker in CLL [72]. Another study has been shown that there were correlations between BCL-2 expression, M4/5 subtypes, WBC-platelet counts, Hb level, complete remission rate, and shorter survival. Moreover, BCL-2 expression had a prognostic value among Iranian AML patients [73]. BECN1 is a regulator of autophagy and cell death through interaction with BCL-2 and PI3K. BECN1 down regulation was associated with FLT3-ITD mutation, higher age, and WBC count in a sample of Iranian AML cases. Moreover, there was an association between reduced expression and shorter RFS in cytogenetically intermediate risk group [74]. The association between myeloid antigens (CD13 and CD33) and BCL-2 were also assessed in a sample of Iranian ALL cases. It was observed that there were significant correlations between BCL-2, myeloid antigents expressions, and survival. Simultaneous expression of BCL-2 and myeloid antigens was correlated with a poorer prognosis [75]. The RASSF (Ras association domain family) is a family of proteins that have tumor suppressor function. RASSF6 is a proapoptotic factor through caspase independent or dependent pathways. The frequency of RASSF6 and RASSF10 promoter methylations and their correlation with overall survival and clinical parameters were assessed in Iranian ALL cases. They showed that the RASSF6 methylation status was significantly assoctiated with a poor prognosis and a shorter overall survival in patients with pre-B-ALL which suggested the epigenetic regulation of RASSF6 during ALL progression [76].

Poly ADP-ribose polymerase-1 (PARP-1) is a key factor in DNA repair [144]. It has been found that the PARP-1 mRNA expression was significantly increased among Iranian non-M3 AML patients compared with healthy controls. There were also correlations between PARP-1 over expression, worse overall survival, and relapse-free survival. Therefore, PARP-1 over expression can be introduced as a poor and independent prognostic factor in non-M3 AML [77]. Another study has been observed that there was an increased level of PARP1 expression among a sub population of Iranian AML patients compared with controls. PARP1 was up regulated in AML patients with poor prognosis in comparison with good or intermediate prognosis. Furthermore, PARP1 was over expressed in patients with chromosomal translocations compared with those without chromosomal translocations [78]. MTHFR is a critical enzyme during folate metabolism, which catalyzes the irreversible conversion of 5, 10 methylenetetrahydrofolate (5, 10-MTHF) to 5-Methyl THF. It is involved in DNA repair and methylation [145]. It has been reported that the AC heterozygous genotype of rs1801131 (A1298C) polymorphism significantly decreased the risk of ALL in comparison with AA homozygous genotype. Both rs13306561 TC and TC + CC genotype significantly decreased the risk of ALL compared with TT genotype. The C allele decreased the risk of ALL in comparison with T allele. MTHFR rs1801131 and rs13306561 polymorphisms decreased the risk of ALL in a sample of Iranian population [79]. The serine hydroxymethyltransferase 1 (SHMT1) catalyzes the serine hydroxymethyltransferase reaction during the methionine and purines synthesis which are essential in DNA synthesis and repair [146]. The probable association between SHMT1 polymorphisms and childhood ALL was assessed in a sample of Iranian patients. It was observed that the rs9901160, rs2273027, and rs1979277 polymorphisms significantly increased the risk of childhood ALL. However, rs9909104 polymorphism significantly reduced the risk of ALL. Therefore, polymorphisms of SHMT1 gene were correlated with childhood ALL risk in a sample of Iranian population [80]. The fragile histidine triad (FHIT) is an adenylohydrolase involved in purine metabolism. The prevalence of FHIT hypermethylation was significantly higher in a sample of Iranian ALL cases compared with controls. Therefore, epigenetic regulation of FHIT can be associated with pediatric ALL progression [81].

CDKN2B is a cyclin-dependent kinase inhibitor that suppresses G1 cell cycle progression. Promoter methylation of CDKN2B was evaluated in Iranian AML patients. The highest *CDKN2B* promoter methylation incidence was observed among AML- M2, whereas the M3 and M4 subtypes had lower incidences of methylated CDKN2B. Patients with methylated CDKN2B showed higher survival compared with those without methylation. Therefore, CDKN2B promoter methylation can be considered as potential prognostic factor for the survival prediction of Iranian AML patients [82]. Telomeres are repeated DNA sequences at the chromosomes ends that have a critical role in chromosomal stability. The progressive telomeres shortening and telomerase activation have been considered as the key mechanisms in chromosomal integrity and tumor progression. It has been observed that the majority of Iranian APL patients had a significant reduction in telomere length (TL) compared with controls. Moreover, there was a significant association between TL and PML-RARa expression. Telomerase was also activated in all patients whereas the TA level was significantly higher among relapsed patients compared with new diagnosed patients. The shortened TRF and increased TA were significantly associated with poorer overall survival [147].

### Signaling pathways and transcription factors

WNT is an important developmental signaling pathway associated with tumorigenesis [148–150]. Wnt signals target cells via frizzled receptors (Fz-R). In the canonical WNT pathway, β-catenin enters into nucleus and consequently activates transcription of target genes involved in cell proliferation, cell death, and cell–cell communication [151]. The non-canonical Wnt/Ca^2+^ pathway also inhibits the canonical pathway by CCND1 down regulation. It has been observed that there were significant WNT-7B, WNT-9A, and WNT-16B up regulations, whereas WNT-2B, WNT-5A, WNT-7A, and WNT-10A down regulations among a sub population of Iranian B-ALL cases. Moreover, there were significant decreased expressions of WNT-2B, WNT-5B, WNT-7A, WNT-10A, and WNT-11 among T-ALL cases [83]. Another study reported that there were WNT-3, WNT-10A, WNT-4, WNT-7B, WNT-5B, WNT-9A, and WNT-16B over expressions in Iranian CLL patients compared with normal cases. The WNT-7A expression level was lower in CLL patients compared with controls. There was also a correlation between IgVH mutation and WNT genes in which the patient with unmutated Ig VH genes had WNT-5A and WNT-9A over expression compared with mutated Ig VH carriers. Moreover, there were significant WNT-3 and WNT-9A under expressions in VH3 positive patients compared with VH1 or VH4 positive cases [84]. Another study has been also reported that there were significant up regulation of WNT-3 and down regulations of WNT-7A and Wnt-10A among a group of Iranian AML compared with normal cases [85]. Secreted frizzled-related proteins (SFRPs) are WNT antagonists which suppress WNT signaling pathway in normal state. Methylated SFRP genes lose their inhibitory effect on WNT pathway leading to cell cycle deregulation and leukemogenesis of AML. It has been shown that the methylation of SFRP1 and SFRP2 were observed in all FAB subgroups of AML except M6 in a sample of Iranian subjects [86]. Hippo pathway is associated with organ size by regulation of cell growth and apoptosis. The SAV1, MST, and LATS family members finally phosphorylate the YAP. LATS2 is involved in regulation of cell cycle through inhibition of cyclin E/CDK2 and CDC2. The LATS2 over expression has been reported in a sample of Iraninan AML patients [87]. Ribosomal 6 kinase (*RSK4*) is a tumor suppressor downstream of ERK/MAPK signaling pathways. It has been reported that there was a significant down regulation of RSK4 in a sample of Iranian AML patients compared with non-AML group. Moreover, there was a significant *RSK4* down regulation in AML with t (15;17) compared with other translocations. Therefore, the ERK/MPAK pathway activation increases leukemogenesis and results in poor prognosis in AML cases [88].

CEBPA is a bZIP transcription factor which form heterodimers with CEBP-beta and CEBP-gamma, and c-Jun. It suppresses cell cycle through CDK2 and CDK4 inhibitions. Moreover, CEBPA is associated with normal mature granulocyte formation and AML progression [152]. RUNX1 is also a transcription factor involved in differentiation of hematopoietic stem cells to the mature blood cells [153]. It has been reported that there were significant over expressions of CEBPA and RUNX-1 among a sub population of Iranian AML patients. M3 subtype showed the highest and M4/M5 subtypes showed the lowest expressions for CEBPA. The highest and lowest RUNX1 expressions were observed in M0/M1/M2 and M3 subgroups, respectively [89]. The t (12;21) (p13;q22) chromosomal translocation is very common in pediatric B-lineage ALL which leads to the fusion of RUNX1 and ETV6. ETV6/RUNX1 is one of the most frequent gene fusions among Iranian childhood ALL cases [154]. The Wilms tumor 1 (WT1) is a zinc finger transcription factor that is associated with normal development of urogenital system. WT1 is an important regulator of normal and malignant hematopoiesis and cellular apoptosis. It has been shown that there was significant WT1 over expression in AML patient which introduced WT1 gene expression as a molecular marker for hematopoietic malignancies [90]. The WT1 expression and serum levels of IL-12 and C3 complement were assessed among Iranian acute leukemia patients compared with healthy controls. It was shown that there was significant WT1 over expression, while significant decreased serum levels of IL-12 and C3 in acute leukemia patients compared with controls. The levels of WT1 expression were conversely correlated with serum levels of IL-12 and C3. WT1 may decrease serum levels of IL-12 and C3 in Iranian acute leukemia patients [91]. Another study has been shown that the WT1 was expressed in leukemic cells of newly diagnosed and relapsed patients among a sub population of Iranian cases. The levels of WT1 expression were significantly higher in newly diagnosed and relapsed ALL patients compared with ALL patients at remission and normal cases [92]. Ikaros family zinc finger 1 (IKZF1) is a critical transcription factor during hematopoiesis and lymphoid differentiation. It has been observed that the rs11980379 T > C, rs4132601 T > G, and rs10272724 T > C polymorphisms significantly increased the ALL susceptibility among Iranian population [93].

### Tyrosine kinases and growth factors

FLT3 is a member of the class III receptor tyrosine kinase (RTK) family which has critical roles in regulation of hematopoietic cells proliferation and differentiation. The activating mutations in FLT3 include an internal tandem duplication (FLT3-ITD) in JM domain and a point mutation at codon 835 in TK domain. Nucleophosmin encodes NPM1 that is a shuttle chaperone between the nucleus and cytoplasm. Several functions have already been described for NPM1 protein such as regulation of centrosome duplication and ribosomal function. It has been observed that the frequencies of FLT3-TKD, FLT3-ITD, and NPM1 mutations were higher in Iranian CN-AML patients compared with AML subjects with cytogenetic anomalies. Therefore, NPM1 and FLT3 can be used to monitor CN-AML patients, especially for MRD [94]. Another study has been shown that there was a high frequency of FLT3-ITD mutation among Iranian AML cases [95]. It has been shown that there was a high frequency of NPM1 mutations in the monocytic of Iranian AML cases which were also correlated with FLT3/ITD mutations [96]. There was a significant correlation between FLT3 mutation and M3 subtype, while the KIT mutation was significantly correlated with M2 and M4 subtypes [97]. ZAP70 is a member of the Syk tyrosine kinase family. It has been shown that there were significant up regulations of ZAP70 expression in Del11q13 and del17p13 subgroups in comparison with control cases among a sub population of Iranian B-CLL subjects [98]. Another study has been reported that the levels of ZAP70 expressions in Iranian del13q B-CLL subjects were similar to normal controls, whereas it was increased in del11q and trisomy 12 subgroups [155].

The orphan RTK (ROR1) is a member of RTK family. It has been reported that there were ROR1 and WT1 up regulations mainly in mature and immature subsets of Iranian B-ALL patients, respectively. It was suggested that the expressions of ROR1 and WT1 were correlated with differentiation of leukemic cells in B-ALL cases [99]. Vascular endothelial growth factor (VEGF) is one of the critical regulators of angiogenesis. The VEGF-A and VEGF-C are expressed by AML cells and associated with leukemic cell proliferation and resistance to chemotherapy. The expressions of VEGF-A, VEGF-C, and MVD were assessed in Iranian AML patients before and after chemotherapy. It was shown that there was a reduction in angiogenesis after chemotherapy. The anti-angiogenic drugs (e.g. thalidomide) and chemotherapy were considered effective in the treatment of AML [156]. Another study has been reported that there was significant decreased levels of VEGF-C expression among a sub population of Iranian AML patients compared with healthy controls [100].

### Cytoskeletal factors and cancer testis antigens

Brain and acute leukemia-cytoplasmic (BAALC) is associated with regulation of actin cytoskeleton and lipid rafts [157]. It has been reported that the BAALC was significantly up regulated in a sample of Iranian AML patients, especially in M1 and M2 subtypes. Moreover, BAALC up regulation was associated with poor prognosis [101]. It has been shown that the T-ALL carriers of G424T (rs62527607) polymorphism (TT or GT genotype) had a lower chance of treatment compared with pre-B-ALL among a group of Iranian cases. Whereas, T-ALL and pre-B-ALL carriers of GG genotype had similar survival rates. The rs62527607 polymorphism was introduced as a negative prognostic marker among Iranian patients [102]. The prognostic significance of the BAALC gene expression levels and its association with MDR1 were also evaluated in Iranian pediatric ALL. It was shown that the BAALC overexpression was a negative prognostic factor in childhood ALL. There was a direct correlation between BAALC and MDR1 expressions. Simultaneous BAALC and ABCB1 overexpression in MRD+ ALL children may activate a mechanism in which the BAALC adversely affects the response to treatment [158].

Testis-specific gene antigen (TSGA10) is a cancer testis antigen associated with spermatogenesis [159]. Cancer-testis (CT) antigens have expression in normal testis and tumor cells which introduced these factors as efficient targets for the tumor immunotherapy [160]. It has been observed that there were TSGA10 expressions in majority of bone marrow samples and all peripheral blood samples among a group of Iranian ALL subjects [103]. HIF-1α is a transcription factor and master regulator of cell response to the hypoxia [161]. It has been reported that there were significant decreased expression of TSGA10 and increased expression of HIF-1α among a sample of Iranian AML patients compared with normal controls. There was also a significant inverse correlation between expression levels of TSGA10 and VEGF genes in AML patients. Up regulations of HIF-1α and VEGF in AML patients can be associated with reduction of TSGA10 which is inhibitor of HIF-1α [104].

## Conclusion

In present review we have summarised all of the genes involved in blood malignancies which have been reported among Iranian population. To clarify the molecular biology of blood malignancies, we categorised all of reported genes based on their cell and molecular functions. It was observed that the epigenetic and immune factors were the most common molecular processes associated with progression of blood malignancies among Iranian population. This review paves the way to introduce a diagnostic population based panel of genetic markers for the early detection of blood malignancies among Iranians and also can be performed similarly in other populations.

## Data Availability

The datasets used and/or analyzed during the current study are available from the corresponding author on reasonable request.

## Abbreviations

ALL: Acute lymphoblastic leukemia
AML: Acute myeloid leukemia
CML: Chronic myeloid leukemia
CLL: Chronic lymphoblastic leukemia
HL: Hodgkin Lymphoma
NHL: Non-Hodgkin Lymphoma
MM: Multiple myeloma
lnc-RNAs: Long non-coding RNAs
nc-RNAs: Non-coding RNAs
PAX8-AS1: PAX8 antisense RNA 1
miRNAs: MicroRNAs
HSCT: Hematopoietic stem cell transplantation
IFN-γ: Interferon gamma
CSF: Colony-stimulating factor
PD-1: Programed death 1
ICOS: Inducible T-cell costimulator
DLBCL: Diffuse large B-cell lymphoma
FL: Follicular lymphoma
LT-α: Lymphtoxin-α
MHC: Major histocompatibility complex
NK: Natural killer
KIR: Killer cell immunoglobulin-like receptors
BMT: Bone marrow transplantation
aGVHD: Acute graft-versus-host disease
FCRL: Fc receptor-like
ABCC4: ATP-binding cassette subfamily C member 4
HO-1: Heme Oxygenase-1
GSTs: Glutathione S-transferases
APAF-1: Apoptotic protease activating factor 1
PARP-1: Poly ADP-ribose polymerase-1
SHMT1: Serine hydroxymethyltransferase 1
FHIT: Fragile histidine triad
TL: Telomere length
SFRPs: Secreted frizzled-related proteins
IKZF1: Ikaros family zinc finger 1
RTK: Receptor tyrosine kinase
BAALC: Brain and acute leukemia-cytoplasmic
CT: Cancer-testis
